# Developing an adaptive paediatric intensive care unit platform trial with key stakeholders: a qualitative study

**DOI:** 10.1136/bmjopen-2024-085142

**Published:** 2025-01-07

**Authors:** Tracy Karen Mitchell, Julie C Menzies, Padmanabhan Ramnarayan, Doug William Gould, Elizabeth Deja, Shelley Marsh, Jennifer Ainsworth, Jennifer Preston, Hannah Sedgwick, Carly Tibbins, Paul R Mouncey, Mark J Peters, Kerry Woolfall

**Affiliations:** 1 Public Health, Policy and Systems, University of Liverpool, Liverpool, UK; 2 Paediatric Intensive Care Unit, Bristol Royal Hospital for Children, Bristol, UK; 3 Department of Surgery and Cancer, Imperial College London, London, UK; 4 Intensive Care National Audit and Research Centre, London, UK; 5 Institute of Population Health, Department of Public Health, Policy and Systems, University of Liverpool, Liverpool, UK; 6 Patient and Public Partner, London, UK; 7 GenerationR Liverpool Young Person’s Advisory Group, Alder Hey Children's Hospital Clinical Research Facility, Liverpool, UK; 8 NIHR Clinical Research Network West Midlands, Birmingham, West Midlands, UK; 9 NIHR Biomedical Research Centre, Great Ormond Street Hospital For Children NHS Trust, London, UK; 10 Institute of Child Health, University College London, London, UK

**Keywords:** QUALITATIVE RESEARCH, Clinical Trial, Paediatric intensive & critical care, Randomized Controlled Trial, PAEDIATRICS, Adolescent

## Abstract

**Abstract:**

**Objectives:**

Platform trials were used successfully in adult populations during the COVID-19 pandemic. By testing multiple treatments within a single trial, platform trials can help identify the most effective treatments (and any interactions between treatments) for patients more quickly and with less burden for patients and their families. The aim of this qualitative research was to inform the design of the first adaptive platform trial for paediatric intensive care in the UK with young people, parents/carers and paediatric intensive care unit (PICU) staff.

**Design:**

Qualitative semistructured focus group study. Data were analysed using reflexive thematic analysis.

**Participants:**

Young people, parents/carers, and PICU medical, nursing and research staff.

**Setting:**

The UK.

**Results:**

A total of 86 participants (18 young people; 15 parents/carers; 53 PICU staff) took part in 1 of 10 focus groups between May and September 2023. Participants viewed the proposed PICU platform trial and use of research without prior consent to be acceptable. Findings provide insight into how the PICU platform trial should be designed and operationalised, including having a broad and inclusive population eligible for inclusion onto the platform trial, with different inclusion and exclusion criteria for each domain; starting the trial with no more than three domains and prioritising the outcomes of *Child quality of life* and *Survival* (all participants). Optimal governance structure and suggestions about how any challenges to the success of the full trial can be overcome are also presented.

**Conclusions:**

Young people, parents/carers and PICU staff viewed the proposed PICU platform trial to be acceptable. These key stakeholders supported us with the design of an adaptive platform trial for PICU that has a rigorous methodology, yet can be operationalised in a family-centred way, to provide high-quality evidence that can support clinical decision-making and guide the treatment of critically ill children. Our findings have informed the PICU platform trial protocol.

**STRENGTHS AND LIMITATIONS OF THIS STUDY**

- A key strength of this study is that it was designed in collaboration with young people and parents through patient and public involvement and engagement.
- Interview and focus group recruitment was conducted until the point of information power, gaining qualitative insight from all key stakeholders for a future platform trial which included the perspectives of young people and parents with relevant experience as well as paediatric intensive care unit staff.
- A limitation of the study was that, due to the co-development approach, some domains and interventions were not fully defined, and, as a result, the lack of detail made it difficult for some participants to rank their order of priority.
- A further limitation is that due to the focus group format we were unable to use interpreters to engage children and parents who did not speak English.

## Introduction

 Around 20 000 critically ill children are admitted to 1 of 28 paediatric intensive care units (PICUs) in the UK each year,^1^ yet there is a lack of high-quality evidence to support clinical decision-making and guide treatment of these children.^2^ Traditional randomised-controlled trials (RCTs) can be costly, resource-intensive and time-consuming in answering important research questions and translating the findings into practice.^1 3^ This is because each RCT typically addresses a single research question, comparing one or two interventions (medicines, ways of working and/or medical equipment)^4^ with a control and do not have the flexibility to add new questions or interventions later. Traditional RCTs have a specific start and end date.^5^

An adaptive platform trial is an alternative way of conducting RCTs and is designed to allow multiple research questions and interventions to be tested simultaneously within ‘domains’/areas in a single study.^6^ An adaptive platform trial allows for prespecified changes to the trial as it is being conducted,^7 8^ for example, new research questions or interventions can be added in or stopped as the trial progresses.^5 7^ Patient data are examined at multiple time points, which informs these adaptations.^6 7^ Consequently, important questions can be addressed simultaneously.^9 10^ Platform trials such as the Randomised, Embedded, Multi-factorial, Adaptive Platform Trial for Community-Acquired Pneumonia and the Randomised Evaluation of COVID-19 Therapy trial were used successfully during the COVID-19 pandemic^78 11^,^13^ and are currently being developed in a range of other clinical settings and disciplines.^4 14^ To develop the first adaptive platform trial for PICU (to our knowledge), it is important to design the study and its delivery in a way that is acceptable to children, parents/carers (now referred to as parents) and PICU staff, whose views about platform trials are not known.

## Objectives

To conduct qualitative research with key stakeholders (young people, parents and PICU medical, nursing and research staff working in the UK) to inform the design of a large, Bayesian, randomised, multifactorial, adaptive platform trial for paediatric intensive care.

## Methods

### Study design

A qualitative focus group study exploring the practical implementation of the proposed trial, governance structure and population for inclusion with staff; acceptability of new interventions not typically used in PICU, timing of approach and consent processes, and clarity of participant information materials with young people and parents; and the therapy areas (domains) and outcomes of importance and overall acceptability of the proposed trial with young people, parents and staff.

To inform the focus groups, we consulted with the Paediatric Critical Care Society Study Group (PCCS-SG) members, informed by previous research,^15^,^18^ to develop a protocol for responding to distressed participants. We also sought patient and public involvement and engagement (PPIE) to develop draft participant information sheets (PIS) (online supplemental file 1), age-appropriate focus group topic guides (online supplemental file 2) and a potential outcomes list^19^ (online supplemental file 3). Further details regarding this PPIE are outlined below.

**Figure 1:**
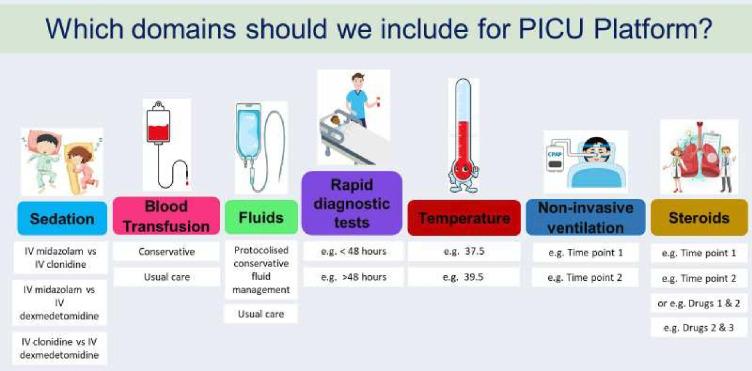
Pre-defined suggestions for domains to include in the PICU-Platform trial. PICU, paediatric intensive care unit.

We developed a presentation for use in focus groups to help explain what a platform trial is (online supplemental file 4), which included seven potential domains for the proposed PICU platform trial which came from a priority setting exercise with PCCS-SG members and coauthors MP and PR in 2022 (figure 1). We used an animation (You Took Part In Research Animation - YouTube)^20^ to help explain the rationale and process for taking a research without prior consent (RWPC) approach due to having little or no time to seek informed consent for critically ill children’s involvement in research.^16 20 21^ An online voting system (Poll Everywhere^22^) was used to collect data about which domains should be included in the platform trial and what outcomes should be measured.

### Patient and public involvement and engagement (PPIE)

PPIE has been central to the planning, design and conduct of this research. The study team established a Parent Advisory Group (PAG) of nine parents of children who had experienced admission to PICU and had experience of PPIE or trial participation. We also worked with six National Institute for Health and Social Care Research (NIHR) GenerationR^23^ Bristol Young Persons Advisory Group (YPAG) members (aged 13–21 years). PPIE consisted of discussions about participant eligibility criteria, recruitment strategies and methods of engagement for the focus groups, as well as participant-facing information and supporting research materials.

Recommendations for the focus groups from both the PAG and YPAG included recruitment of young people from other GenerationR^23^ YPAGs (Birmingham/Liverpool, UK); recruitment of parents who had participated in PICU trials invited from trial databases and through advertisement on social media; offer both face-to-face and online focus groups; consider focus group constitution and implement strategies to aid communication and facilitate inclusion.

Both PPIE groups commented on the content and text of participant-facing information, reducing text, improving clarity and phrasing and advising on images. The number and length of questions within interview schedules and preparatory material about platform trials were also revised to improve ease of understanding.

### Eligibility, recruitment and sampling procedure

Based on previous studies,^15 24 25^ we anticipated conducting six focus groups (two young people, two parent, two PICU staff), each with 6–8 participants. We used existing databases and networks (NIHR GenerationR YPAG coordinators; PCCS-SG members and PICU staff; charities and parent support groups) and online advertising (websites, Twitter and Facebook) to recruit: young people who were members of the GenerationR Liverpool and Birmingham YPAG; parents who were PPIE co-applicants or have/had a trial oversight role on a PICU-based clinical trial steering or trial management group (TMG); parents with experience of having a child admitted to PICU and/or had been recruited to a paediatric research study in the last 3 years; and research-active PICU clinicians, including medical, nursing and research-delivery staff.

Focus groups were limited to those who could speak English.

### Screening and conduct

Expressions of interest were confirmed for eligibility by TKM (PhD, research associate, research methodologist, female). Following this, prospective participants were sent the draft platform trial PIS, consent form and potential outcomes list to read prior to the focus groups.

Written or audio-recorded verbal consent was obtained from adult participants. Young people provided written consent/assent. Parents of young people under 16 years provided written or audio-recorded verbal consent for their child to take part. TKM, KW (PhD, professor, social scientist, female, qualitative study lead investigator) and ED (PhD, research fellow, psychologist, female) facilitated focus groups. Digital audio recordings were transcribed verbatim by UK Transcription,^26^ then anonymised and checked for accuracy by TKM. Recruitment stopped when the point of information power^27^ and variance (inclusion of all groups and sample diversity) was reached. Parents and young people received a £30 voucher to thank them for their time.^28^

### Analysis

TKM led the analysis with oversight from KW. The transcripts were read and reread to note down initial ideas before being imported into NVivo V.14,^29^ which is the software that was used to assist the organisation and coding of data, including the domains and outcomes data collected in Poll Everywhere and exported into Microsoft Excel.^30^ Deductive codes were initially identified from the study protocol and interview/focus group topic guides. Analysis was then broadly inductive, thematic and iterative.^31^,^33^ KW second coded 40% of transcripts and then checked these against TKM’s codes. Transcripts coded before new codes or subcodes were identified were revisited to ensure that the new codes were representative of the data coded under them. All codes and subcodes were interrogated by TKM and KW to search for and name themes that would provide a clear and trustworthy account of the data. TKM and KW met regularly to discuss the names and definitions of codes, subcodes, themes and subthemes, so that they remained nuanced. The names of themes and subthemes were agreed on by all authors. A weighted point-based system was used to determine the top prioritised outcomes, the importance of which has been highlighted in other studies.^2434^,^36^ As there were 16 predefined outcomes, a score of 16 was given to the outcomes that participants ranked most important, a score of 15 for the outcomes ranked second most important, and so on, down to the least important (16th) outcome being given a score of 1. Findings were fed into an NIHR Health Technology Assessment (HTA) Programme funding application.

The Consolidated Criteria for Reporting Qualitative Research checklist^37^ was used to aid reporting (online supplemental file 5).

## Findings

### Participation

Forty PICU staff took part in one of four focus groups that explored population (PICU staff, FG1), initial trial domains (PICU staff, FG2), outcomes (PICU staff, FG3) and governance (PICU staff, FG4) at the PCCS-SG face-to-face meeting in May 2023. We had anticipated conducting one focus group at this meeting, but due to large numbers, we conducted four separate focus groups lasting between 34 and 41 min. A further 46 participants (18 young people, 15 parents and 13 PICU staff) took part in one of six subsequent focus groups between May and September 2023, lasting between 67 and 100 min (see table 1). An NIHR GenerationR YPAG coordinator was present for both young people focus groups to put the young people at ease and aid communication. Two parents who had agreed to attend the first parent focus group could not attend due to family emergencies.

**Table 1:** Participant demographics

Date	Identifiers	Participant type, format and length of focus group	Number and demographics of participants
13 May 2023	YP1-9, male/female, FG1	**Young person focus group 1** (face-to-face, Birmingham); 70 min	9 (four males; five females), age 14–17 years
5 July 2023	YP1-9, male/female, FG2	**Young person focus group 2** (online); 70 min	9 (six males; three females), age 12–19 years
17 May 2023	PICU staff, FG1 (population)	**Staff focus group 1** at PCCS-SG meeting (face-to-face, Liverpool); 34 min	12 (five medical and six nursing staff)
17 May 2023	PICU staff, FG2(domains)	**Staff focus group 2** at PCCS-SG meeting (face-to-face, Liverpool); 41 min	9 (three medical and six nursing staff)
17 May 2023	PICU staff, FG3 (outcomes)	**Staff focus group 3** at PCCS-SG meeting (face-to-face, Liverpool); 35 min	9 (four medical and five nursing staff)
17 May 2023	PICU staff, FG4 (governance)	**Staff focus group 4** at PCCS-SG meeting (face-to-face, Liverpool); 41 min	10 (three medical and seven nursing staff)
5 July 2023	S1-9, PICU staff, FG5	**Staff focus group 5** (online), representing six sites; 83 min	9 (three medical and six nursing staff)
17 August 2023	S1-4, PICU staff, FG6	**Staff focus group 6** (online), representing four sites; 67 min	4 (three medical and one nursing staff)
6 July 2023	P1-7, mother/father, FG1	**Parent focus group 1** (online); 69 min	7 (three fathers; four mothers)
6 September 2023	P1-8, mother/father, FG2	**Parent focus group 2** (online); 100 min	8 (four fathers; four mothers – one bereaved)

PCCS-SGPaediatric Critical Care Society Study GroupPICUpaediatric intensive care unit

### Approach to consent for the PICU platform trial

After explaining the rationale and process for seeking consent in emergency situations and showing the animation, participant views were sought on the acceptability of conducting the PICU platform trial using a RWPC approach. With the exception of two young people who felt that informed consent should be gained from parents before children are entered into the trial, all other young people, parents and staff *‘were fine with’* (P1, father, FG2) the proposed RWPC approach in time critical situations, with the caveat that parents be informed *‘at the first opportunity’* (P7, mother, FG2) after the *‘life-threatening situation’* (P3, father, FG2) has passed. Staff described this as *‘a good approach… [that] seems to be supported by parents’* (PICU staff, FG6) and has worked well in other PICU studies.

### Timing of approach ‘is key’

Parents emphasised that they were *‘very much in a state of shock’* when their child was admitted to PICU (P6, mother, FG1) and suggested that staff check with parents *‘that they’re in a position, at that point, to receive that information’* (P2, mother, FG2). They stressed the importance of ‘*trying to find the right time to give’* trial information to parents (P5, mother, FG1) for them to be able to *‘take it all in’* and said that *‘timing … that delivery and the approach of the parents … is key’* (P7, father, FG1).

While some young people and parents said that they would want to be given information about the PICU platform trial *‘as soon as possible’* (P2, mother, FG2), *‘even in the most stressful situation’* (P4, mother, FG2), other parents said that they would not want to be approached until their child was *‘stabilised’* (P1, father, FG2; P4, mother, FG2). These findings were in line with staff who emphasised the need for flexibility as you can’t *‘put a set time on… [approaching families], it is dependent on the individual families’* (PICU staff FG5) but added that *‘the first day is never a good day for a family’* (PICU staff, FG5).

### Consulting with parents about research participation

Although one young person said *‘that the child should be told [about their involvement in the trial], no matter what their age is’* (YP9, female, FG2), there was agreement among young people and parents that whether or not children should be informed about participation in the trial during their PICU stay *‘depends on the child’* (P5, mother, FG2) and ‘*how poorly they are’* (P2, mother, FG2). As some children might become ‘*more anxious’* (P2, mother, FG2) or *‘upset’* (P5, mother, FG2) by being informed, *‘parents should be spoken to first’* (YP4, female, FG2) because it is a parent’s ‘*responsibility to make that decision’* (P1, father, FG2) and parents will ‘*know how a child’s going to react to a certain amount of information’* (YP8, male, FG2). If a decision was made to provide information, then young people and parents suggested that older children ‘*should be told in more depth… that they are part of a trial… [once they are well enough] …, younger [children from age 5 years] … more briefly’* (YP9, female, FG2).

### Information delivery: one size does not fit all

Young people and parents were asked to review the PIS for the PICU platform trial (online supplemental file 1). Although they viewed the PIS as clear *‘in the way that it’s laid out’* (YP6, male, FG2) and liked the *‘images and the bright colours’* (YP5, female, FG2), some parents found the PIS to be *‘very wordy’* and said that it could *‘overwhelm’* parents (even with only three domains included) (P5, mother, FG1).

Many young people and parents emphasised the importance of using ‘*different formats’* (P5, mother, FG2) to raise awareness about the trial including the PIS; posters in the PICU; a study website (also accessed via a *‘QR code’* (YP7, FG1)); and videos and PDF files on social media sites such as *‘TikTok, Instagram … Facebook’* (P2, mother, FG2), *‘Snapchat… and YouTube’* (P4, mother, FG2). However, face-to-face discussions with an ‘*approachable, friendly’* person from the research team (P2, mother, FG2) who has a *‘compassionate manner’* (P4, mother, FG2), and having the contact details for that person to answer any questions *‘that come up later… after you’ve consented’* (P6, mother, FG1) was viewed as being the most important (online supplemental file 6). Families valued being given time (at least overnight) to reflect on trial information before being reapproached for a consent decision.

### Prioritisation of outcomes

After being provided with an explanation about what outcomes are, all participants were asked to look at the provided list and consider if any important outcomes were missing for the PICU platform trial. The additional outcomes suggested are shown in box 1. Several edits to outcomes were suggested by all participant groups, especially for *Child quality of life* and *Family quality of life* (online supplemental file 7).

Box 1 **Suggested outcomes that are missing for the PICU platform trial**

- Health economics/health service resources used—cost to healthcare
- The one that will establish utility or futility of the intervention
- Location at hospital discharge
- Location at 30 days post event (hospital/home/hospice/mortuary, etc)
- Population incidence (eg, A. Microbial resistance)
- Nutrition, energy targets or weight
- Staff—retention/satisfaction; facilitation of better relationships with families and staff; engagement and motivation; stewardship
- Family experience of care
- End of life—processes; quality of experience; place of death

*Child quality of life* and *Survival* were ranked in the top three outcomes of importance by all participants (online supplemental file 8). Parents and young people also ranked *Adverse events* and *Family quality of life* as being the most important outcomes to measure, although that *Child quality of life* and *Family quality of life* are ‘*overlapping’* (YP9, female, FG2) and linked with *Overall length of the child’s hospital stay(s*) and should be measured long term:

If there’s a better chance of a better quality of life and survival or less side effects, I think that’s something you should consider because this child is only a child and it could affect them forever. (YP6, female, FG1).

Parents also prioritised *Symptoms of medical condition(s), disease(s) or infection(s); Type of intervention (support/treatment/medication) given, and when*; and *Number and type of child’s organs that required support while in PICU/hospital*. Staff from all groups ranked outcomes to do with time as being most important to measure for the PICU platform trial (*Duration of organ support; Readmission to PICU/hospital within a certain time period* and *Number of times admitted to PICU during the hospital stay*); young people and parents did not prioritise these.

Some staff said that outcomes need to be developed, defined and carefully selected once the domains and population have been decided for the trial, so that they are relevant for multiple domains. They said that some outcomes should be overall outcomes for the PICU platform trial, and some will be domain and population specific.

### Prioritisation of domains for the PICU platform trial

All participants were shown a presentation with the seven domains suggested for the PICU platform trial (figure 1) and asked what they thought about them, whether there were any missing domains and which domains are important to include in the platform trial.

The domains that staff felt were most important to include in the PICU platform trial were *Fluid management* and *Sedation management*, with the other five domains being less of a priority for the start of the trial yet considered suitable for inclusion in the future. Parents, on the other hand, prioritised *Rapid diagnostic tests* and *Non-invasive ventilation*. Young people recommended using domains that are interlinked, for example, *Fluid management* and *Temperature management; Rapid diagnostic tests* and *Steroids*; and *Sedation management* and *Non-invasive ventilation*.

Parents and staff stated that additional information was needed about the nature of the intervention/‘drug’ or ‘time point’ for other domains to establish the logistics and acceptability of each particular domain. Some parents stated that it is difficult to prioritise domains without knowing the risks of any interventions within the domains to assess whether they would want their child to take part in that domain or not. Explaining the domains and the risks of interventions *‘would make a big difference’* to parents (P4, mother, FG2).

Suggestions for additional domains included *‘antibiotic use’* (P7, father, FG1)/*‘antimicrobial stewardship… - the incidence of multidrug resistant organisms’* (PICU staff, FG3) and nutrition practices. Staff suggested building biobanking and sample collections into the platform trial in the future.

### Keep the number of domains ‘simple and small … to start with’

All participants were asked how many different domains would be acceptable for children to be included in at any one time. While a few parents said that the maximum number of domains used in the trial would not matter and should not be limited, so long as they were *‘legitimate areas of research’* (P1, father, FG2) and *‘presented clearly [to parents]’* (P1, father, FG1), other parents, young people and staff stated that *‘three … [domains] would be plenty to start with’* (PICU staff, FG5). More than three domains were viewed as being *‘difficult to show to parents’* (PICU staff, FG5) and would potentially lead to *‘information overload’* (PICU staff, FG6) and parents feeling overwhelmed, as well as stretching research staff capacity.

### Participation in domains

Providing parents with the option to ‘opt out’ of individual domains was viewed as *‘really important’* (YP6, female, FG1), enabling parents who were worried about particular domains to feel *‘more comfortable’* (YP8, male, FG1) about the concept of a platform trial design. Having a more flexible approach for staff to ‘opt out’ of involvement in a particular domain would also enable PICUs who *‘didn’t use that drug or weren’t happy with’* a specific domain (PICU staff, FG5) to participate. Many staff felt that the trial would *‘lose families’* (PICU staff, FG5) and not maximise *‘the number of units’* taking part (PICU staff, FG5) *‘if an all or nothing’* approach is taken (PICU staff, FG5).

### Types of intervention within domains

While one young person said that children *‘should only get tried and tested medicine’* (YP1, male, FG2), parents and staff said that they would be happy with including treatments used in neonatal intensive care or adult settings, but not new ‘untested’ treatments. Parents suggested explaining the *‘pro and cons’* of interventions new to PICU clearly (P4, mother, FG2) and ‘*why they want to try it and where it was successful before’* (P1, father, FG1). The nature of the intervention itself was regarded as important, including *‘how new the intervention is, and if a normal intervention is being used in a new way, which it’s not used in now’* (PICU staff, FG6). One group highlighted the need for agreement from their PICU colleagues, but *‘in principle it should be okay if the whole unit’s agreeable’* (PICU staff, FG5).

Some PICU staff had concerns about the use of Dexmedetomidine (an ‘α2 adrenergic receptor agonist… used for procedural sedation or anaesthetic premedication in children’ ^38^ (pp. 12)) and Midazolam (a benzodiazepine which is a common sedative used in children^38^) in the sedation domain as they did not use this drug at their PICU or would not want to use it for children with cardiac conditions. Staff in focus group 1 suggested that each domain could have a different inclusion and exclusion criteria, which might address such concerns. Young people discussed interactions between drugs/treatments/interventions used within the different domains and asked questions about what would happen if a child had an allergic reaction to a medicine:

I suppose this is medicine dependent, but if the medicine has a lot of things that people are commonly allergic to…, how would we know that, if it’s in an emergency context, if we shouldn’t give it to that patient? (YP6, male, FG2)

### Population for inclusion

Staff were asked who the target patient group for inclusion in the PICU platform trial should be. Overall, there was staff agreement that the PICU platform trial should be *‘as inclusive’* (PICU staff, FG5) and *‘as broad as possible’* (PICU staff, FG2) *‘at that top level’* (PICU Staff FG5), as shown in table 2.

**Table 2:** PICU platform trial inclusion criteria suggestions

All admissions to PICU	Including ‘looked after children (who) are often not included in research’ (PICU staff, FG1).
	Including children who are palliative or who need cardiac care (PICU staff, FG1).
All admissions requiring organ support	Organ support requires a definition ‘and to be really carefully… consulted a bit more’ (PICU Staff, FG6).
‘All ventilated or non-invasively ventilated patients’	‘So, from low flow, nasal canula, all the way through to intubation’ (PICU staff, FG1)

PICUpaediatric intensive care unit

Stratum (predefined subgroups of patients who may influence trial outcomes) identified by staff for the PICU platform trial included children with cardiac conditions, traumatic brain injury, cancer, multiple morbidities and in receipt of palliative care: Age and planned/unplanned admission were also considered. Illustrative quotations are shown in online supplemental file 9.

There was agreement across staff focus groups that having a different inclusion and exclusion criteria for each domain would mean that the trial could generate evidence about clinical decision-making for specific populations such as ‘*cardiac patients on inotropes’* (PICU staff, FG5) or children with *‘traumatic brain injury… [who] need particularly specific management’* (PICU staff, FG6) depending on the nature of the intervention. There were three main suggestions for exclusion criteria: (1) children who are only on PICU (or high dependency unit (HDU) level care in some hospitals) because *‘they can’t go anywhere else’* (PICU staff, FG1), for example, *‘somebody who, for service delivery reasons, are on the ICU but actually don't need ICU in any other way’* (PICU staff, FG1)/*‘long-term trache patients…waiting for care packages… [who] live on units for almost a year’* (PICU staff, FG1); (2) children who are *‘known to palliative care for symptom control… [and] this is their last admission’* (S1, PICU staff, FG6) – their *‘death is imminent’* (S2, PICU staff, FG6) and children where *‘it is a life or death situation’* (YP4, female, FG1); and (3) children who are in PICU (or level 3 acute critical care) for over 30 days.

### Governance structure

**Figure 2:**
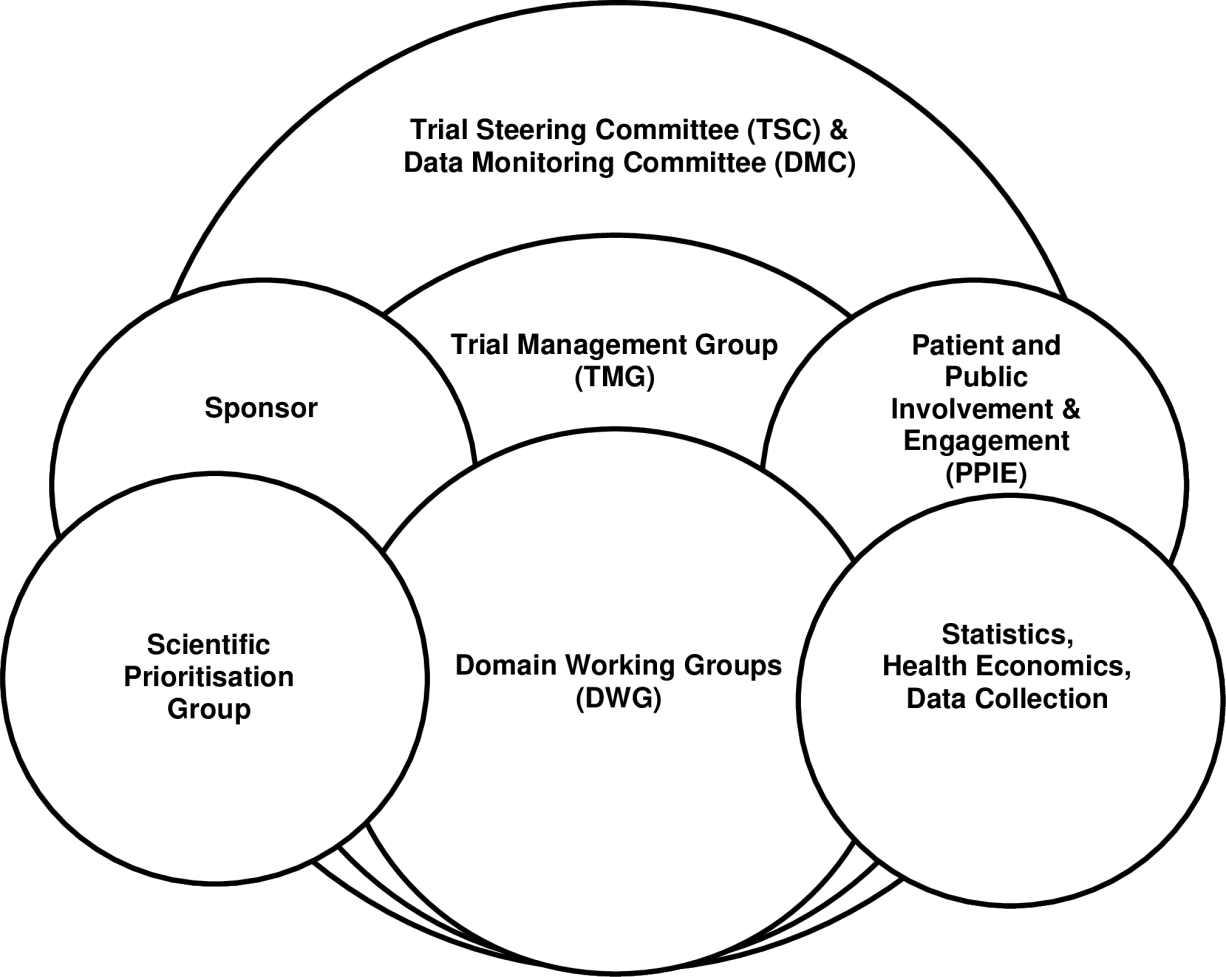
PICU platform trial governance structure proposed by PICU staff. PICU, paediatric intensive care unit.

PICU staff (FG4) recommendations (online supplemental file 10) about how the overlapping governance structure should look for the PICU platform trial (figure 2) were presented to staff (FG 1–3, 5–6), and parents from both focus groups who were asked what they thought about them and who should be involved in making the decisions about adding a new domain to the platform trial.

Several parents and staff spoke about the value of young people, parent and nursing staff *‘on the ground’* (PICU staff, FG4) representation on the TMG and domain working groups (DWGs), as well as for PPIE, to support decision-making about new domains: Parents are *‘somebody who understands it from the other side’* (P2, mother, FG2), while nurses could advocate for patient needs (PICU staff, FG4). One staff member suggested ensuring that all paediatric critical care staff are involved in decision-making, not just PCCS-SG members, because those *‘not actively be involved in PCCS-SG … might have quite strong opinions’* (PICU staff, FG5). Others suggested that the process of setup and governance should be inclusive of all clinical trial units.

Staff recommended that DWG leads are split between an early career and senior researcher. The scientific prioritisation process should include the *‘neutral voices… [of] non-biased’* (PICU staff, FG4) research-active PICU staff from across the world (including low and middle income countries). The importance of having *‘arrangements in place for people to be credited for their intellectual input into the work’* was emphasised by staff (PICU staff, FG6), as was having a process evaluation or study within a trial (SWAT) within DWGs (PICU staff, FG4).

### Acceptability of the PICU platform trial

At the end of each focus group, participants were asked to reflect on their discussions and consider the overall acceptability of the proposed trial. All participant groups were supportive of the proposed PICU platform trial, which they believed would save time and money to *‘provide a lot of valuable evidence’* (P7, mother, FG2) in a *‘more effective [way] than a normal trial’* (YP5, female, FG2):

I think, overall, it’s going to, hopefully, save a lot of time, effort and resource, because interventions will be able to be plugged into the same trial infrastructure. So very supportive, and I think it’s a great idea (PICU staff, FG6).

Parents re-emphasised the importance of communicating trial information clearly, ‘*with conviction and in a suitable way’* (P3, mother, FG1) during recruitment (and later) discussions with parents due to the complexity of the trial design. Parents highlighted the need for transparency about any potential risks associated with child participation and, as mentioned earlier, timing discussions carefully to prevent further burden on parents who were already anxious and stressed about their child.

## Discussion

Young people, parents and PICU staff were supportive of the proposed PICU platform trial and found it acceptable overall, saying it may be a resource and cost-effective way to conduct future clinical trials.^5 6^

An RWPC^3 20^ approach was viewed as being acceptable for time-critical interventions within the PICU platform trial, as found in other studies.^16 24^ There may be a need to seek informed consent or assent (eg, for follow-up data collection), depending on the selection of interventions and outcomes. In line with the Health Research Authority guidance on consent by and on behalf of children and young people,^39^ practitioners should assess a child’s capacity and provide information that is understandable to them even when the child or young person is not deemed competent to make decisions for themselves or when they are not legally required to do so, for example, in a Clinical Trial of an Investigational Medicinal Product (CTIMP).

Danaher *et al*^40^ state that effective communication is critical in healthcare settings for building trust, sharing information and decision-making. Parents and young people highlighted the importance of providing parents (and where appropriate, children) with age-appropriate trial information materials at an appropriate time and in different formats (such as a face-to-face discussion, prerecorded video, animation, participant information leaflet, posters and study website), as recognised in various fields for accessibility, comprehension, engagement and inclusivity reasons.^41^ Consideration should also be given to the environment where this information is given. Our findings suggest that parents should be informed about research participation first and their views sought on how best to tailor and deliver research information for their child once they are stabilised to prevent unnecessary burden or distress (non-maleficence).^42 43^ As highlighted by Habermas’ theory of communicative action,^44^ practitioners should seek to understand parents’ and children’s preferences, emotions and experiences by using individualised communication.

Our findings suggest that the population for inclusion in the PICU platform trial should be broad and inclusive (ie, all admissions requiring PICU-level care) and then have a specific inclusion and exclusion criteria for each domain to account for children with conditions that might influence outcomes. Careful consideration needs to be given regard to the subgroup populations who might influence outcomes (online supplemental file 9).

Participants indicated that the PICU platform trial should start with no more than three domains to keep the amount of trial information manageable for families to make informed decisions about participation. Platform trials can be complex and challenging to operationalise,^45^ and it was evident that ongoing work is needed to develop the proposed domains (eg, the steroid domain) and related interventions so that their acceptability can be fully explored before inclusion, in line with pretrial research and PPIE that would take place for an RCT.

An important finding was that parents and staff should be allowed to opt children out of individual domains, thereby respecting the biomedical ethical principle of autonomy,^42^ as well as a means of optimising trial recruitment and retention.

As suggested for inclusion in the core outcome set for paediatric critical care studies^19^ and found in other work,^17 19 34 46^ all participants viewed the outcomes of *Survival* and *Child quality of life* as the most important for the trial. However, as previously reported,^19^ there was a disparity among different stakeholder groups about other outcomes of priority. *Adverse events* and *Family quality of life* were also prioritised as the most important by parents and young people; *Symptoms of medical condition(s), disease(s) or infection(s*) by parents; and *Length of PICU stay* and *Duration of organ support* by staff. Outcomes will need to be reviewed once population and domains have been decided. The proposed governance structure which highlights the need for collaborative working involving all key stakeholders will help minimise risks to trial success. A pilot phase and ongoing PPIE will help identify and overcome the aforementioned challenges to success.

## Strengths and limitations of this study

Adaptive platform trials are viewed as the potential future of clinical research, and this is the first study to support the design of an adaptive platform trial in PICU. The main strength of this study is undoubtedly the PPIE work conducted with young people and parents and the collaboration with YPAG coordinators and PCCS-SG members to recruit a higher number of participants than is typical for this type of research to inform the design and conduct of this trial. We have also addressed one of the gaps in evidence^2^ by identifying and reporting outcomes of importance for the PICU platform trial.

A limitation of the study was that the domains and interventions within them were not all clearly defined at the time of focus groups as we wished to co-develop them with key stakeholders. Consequently, a lack of detail for a few domains caused some difficulty for participants when trying to rank their order of priority. A further limitation is that due to the focus group format we were unable to use interpreters to engage children and parents who did not speak English. Furthermore, not all children and young people had experience of admission to PICU. Parents, however, all had relevant experience.

## Conclusions

All participants viewed the proposed PICU platform trial and use of RWPC to be acceptable. This qualitative research with key stakeholders has supported the design of this adaptive platform trial for paediatric intensive care. By providing insight into how the trial should be designed and operationalised in a family-centred way, and the population for inclusion, domains/interventions and outcomes of importance and collaborative governance structures to inform the PICU platform trial protocol, any challenges to the success of the full trial can be minimised, especially if a pilot or feasibility study is run first.

## supplementary material

10.1136/bmjopen-2024-085142 online supplemental file 1

10.1136/bmjopen-2024-085142 online supplemental file 2

10.1136/bmjopen-2024-085142 online supplemental file 3

10.1136/bmjopen-2024-085142 online supplemental file 4

10.1136/bmjopen-2024-085142 online supplemental file 5

10.1136/bmjopen-2024-085142 online supplemental file 6

10.1136/bmjopen-2024-085142 online supplemental file 7

10.1136/bmjopen-2024-085142 online supplemental file 8

10.1136/bmjopen-2024-085142 online supplemental file 9

10.1136/bmjopen-2024-085142 online supplemental file 10

## Data Availability

No data are available.
