# Supplementary material for: Developing an adaptive paediatric intensive care unit platform trial with key stakeholders: a qualitative study
Source: BMJ Open. 2025 Jan 7;15(1):e085142. doi: 10.1136/bmjopen-2024-085142 (PMC11749188; doi:10.1136/bmjopen-2024-085142)
Supplement: online supplemental file 1 [file bmjopen-15-1-s001.pdf]

PICU Platform Trial

[NHS hospital logo will be added here]

Information for Parents and Guardians

We would like to invite you and your child to be part of a research study, which is looking at multiple treatment areas within the Paediatric Intensive Care Unit (PICU). Before you decide if you want to give permission for your child to be included in this trial, it is important to understand why the research is being done and what it will involve.

Why has my child been chosen?

Your child is in the intensive care and requires breathing support. Due to the critical nature of their illness, we could not delay giving the urgent treatment your child needed. We have therefore come to talk to you about the study as soon as possible after the medical emergency. This is called "research without prior consent", a method of consent used in other emergency studies.

What happens in this study?

This study is known as a 'platform trial', as it is investigating multiple treatment areas at the same time. Your child may be included in one or more of the areas - the doctors and nurses will discuss this with you. This information sheet contains a section with more detail about each of the treatment areas which are listed below:

Oxygen (see page 3)

Blood pressure (see page 4)

Fluids (see page 6)

Are there any risks or benefits to being in this study?

The risks associated with each treatment area are described in the relevant sections of this information sheet.

We cannot guarantee taking part in this study will directly benefit your child, but the study is looking to find out more about the best way to treat children in PICU in order to improve future care.

Do I have to allow my child to take part?

No, it is entirely up to you whether you allow your child to continue in the study. If you do agree for your child to be on the study, you can withdraw them at any point, without giving a reason. This will not affect the care that either you or your child receives.

What will happen next?

We will explain the study and go through this information sheet with you. If you agree to take part, we will ask you to sign a Consent Form. If your child can understand the research, is happy to take part and can write their name, they will be asked to sign an Assent Form with you, if they want to.

If you decide that you do not want your child to be part of the research, this will not affect the care of you or your child.

We will collect information on your child's treatment and progress on the intensive care unit, the duration of their hospital stay and survival to hospital discharge. More information about the study and how we use your child's data is available from page 7 onwards (**'Further detail about the study'**).

Please take the time to read the following information carefully. Please ask the nurse or doctor who has spoken to you about the study if anything is not clear or if you would like more information.

Oxygen

This part of the information sheet provides further detail about the oxygen treatment area of the study.

What are we trying to find out?

Breathing difficulties are the most common reason for a child to need an emergency admission to intensive care, and a ventilator with extra oxygen is often a vital part of their treatment. We know that adding too much oxygen can injure the lungs and possibly other parts of the body. Current advice is not to use oxygen to achieve ‘usual’ oxygen saturations (98-100%) when the lungs are sick but to aim a little lower (88-97%). Because there isn’t much evidence about what is best, doctors and nurses tend to aim for the higher numbers.

Recent research in adults has found that high oxygen saturations can lead to worse outcomes in emergencies like heart attacks and strokes. We don’t know if this harm is because of the oxygen level itself or a side-effect of the more intensive treatment needed to keep oxygen levels high. Differences in how children’s bodies work compared to adults mean the results of this research cannot be applied to children.

Our aim is to find out whether children who come to intensive care in an emergency who need both ventilation and extra oxygen have better outcomes when doctors/nurses aim for oxygen saturations at the lower end of the recommended range (88-92%), or at levels often currently used (above 94%). Both of these targets are used in standard practice at the moment, but it is not known which is better.

What are the treatments being studied?

In this part of the study, each child will be randomly allocated into one of two groups:

- One group of children receive treatment aiming to maintain oxygen saturations above 94%
- The other group of children receive treatment aiming to maintain oxygen saturation values of 88-92%

Children are put into groups at random by a computer programme. This means your child had an equal chance of being in either group.

Are there any risks?

Using oxygen reduces the effort needed to breathe and increases the oxygen level in the blood. Oxygen is the most common drug used in emergency situations. As with all treatments, there may be complications, but these are rare. Doctors and nurses looking after your child will watch carefully for these.

Blood pressure

This part of the information sheet provides further detail about the blood pressure area of the study.

What are we trying to find out?

There are many treatments used in intensive care to increase blood pressure. These include drugs which make blood vessels narrow and make the heart pump more (“vasoactive drugs”), but these treatments carry risks. Currently to guide these treatments, most doctors aim to achieve a blood pressure in the normal range depending on the child’s age. However, currently there is no clear evidence for the best target to aim for.

We want to find out whether children on intensive care could be managed more safely with lower blood pressure targets, still within the normal range. If this could be shown, it may be that these children could safely receive less drug treatment and they may recover more quickly.

What are the treatments being studied?

In this part of the study, each child will be randomly allocated into one of two groups:

- One group of children receive the intervention, which means they are treated with a lower blood pressure target
- The other group of children are treated with the usual care that they would receive outside of the study

Children are put into groups at random by a computer programme. This means your child had an equal chance of being in either group.

Are there any risks?

In both groups, patients will receive vasoactive drugs due to their condition. Known potential side-effects of vasoactive drugs include:

- abnormal heart rhythms that are not immediately life-threatening
- decreased kidney function
- insufficient blood flow to the intestines
- insufficient blood flow to the limbs, fingers, or toes

All patients will be monitored closely for side effects, as well as any potential side effects associated with lower blood pressure.

If your child is treated in the intervention group (lower blood pressure target)

If your child is in this group, they will receive treatment to keep their blood pressure at the lower target, until they can maintain this target on their own (without needing drug treatment). In theory, targeting a lower blood pressure reduces the amount of vasoactive drugs given. A potential benefit of being in this group is that your child may experience fewer side effects.

However, it is possible that staying with a lower blood pressure target may also present certain risks, similar to those above:

- decreased kidney function
- insufficient blood flow to the intestines or other organs
- insufficient blood flow to the limbs, fingers, or toes

If your child is treated in the usual care group

If your child was assigned to the usual care group, they will be cared for by the hospital's clinical team according to the hospital's current practice.

Fluids

This part of the information sheet provides further detail about the fluid treatment area of the study.

What are we trying to find out?

Septic shock is a life-threatening condition that happens when a child's blood pressure drops to a dangerously low level due to a bacterial infection. Symptoms can include a high temperature, raised pulse rate, quick breathing and confusion. Previously healthy children may develop septic shock, sometimes very quickly. Some children have a higher risk than others, including those with a lowered immune system and those with long-term medical problems.

Across the UK, children showing symptoms of septic shock are treated using fluid bolus therapy. Findings from a recent large African trial suggest that less fluid might be a better in treating children with septic shock than the amount currently recommended. Further research is needed to find out which amount is best for treating septic shock in children.

What are the treatments being studied?

In this part of the study, each child will be randomly allocated into one of two groups:

- Restricted fluid treatment. This group of children will receive 10 millilitres (ml) per kilogram (kg) of their body weight.
- Standard fluid treatment. This group of children will be treated with the usual care that they would receive outside of the study (around 20 millilitres (ml) per kilogram (kg) of their body weight).

Children are put into groups at random by a computer programme.

Are there any risks?

Giving fluid is a standard procedure and the known side-effects are rare. Giving less fluid might help to reduce the chances of these side-effects. The known side-effects of fluid are:

- Allergic reactions (very rare, not serious)
- Nervous system disorders (rare, severe)
- Salt balance disorders (rare and, not serious)

If your child is assigned to the restricted fluid group, they will receive less fluid than is normally given. We believe this approach may be beneficial but we do not know the full risks and benefit, which is why this research is needed.

If your child is assigned to the usual care group, they will receive care in line with the hospital's current practice.

All patients, regardless of which treatment they received, will be monitored closely for side-effects, as well as any potential side-effects associated with restricted fluid. The benefits and risks of different fluid strategies are not known, which is why this research is needed.

FURTHER DETAIL ABOUT THE STUDY

Who is organising and funding this study?

The National Institute for Health Research (NIHR) is funding the study. All NHS research is reviewed by an independent group of people, called a Research Ethics Committee (REC), to protect your interests. This study has been reviewed and approved by the University of Liverpool REC.

The trial is Sponsored by Peter Hyde and is being managed by the Intensive Care National Audit and Research Centre (ICNARC). Members of the team have a lot of experience in caring for children in intensive care and are very active in health research. Parents of children who have experienced being in intensive care have been involved in the development of this study.

What will happen to the results of this study?

The results of this study will be presented at conferences and written up for publication in medical journals, but your child will not be identifiable. We will prepare a summary of the study results for parents who would like a copy. We will also make the results available via the study website and via social media.

How will information about my child be used?

Peter Hyde (ICNARC) is the Sponsor for this study based in the United Kingdom. We will be using information about your child to undertake this study and will act as the data controller for this study. This means we are responsible for looking after your child's information and using it properly.

We will need to use information from your child's medical records and central NHS records for this research project. This information will include your child's name, NHS number and date of birth. People will use this information to do the research or to check your records to make sure that the research is being done properly.

People who do not need to know who you are will not be able to see your child's name or contact details. Their data will have a code number instead.

We will keep all information about you and your child safe and secure.

Once we have finished the study, we will keep some of the data so we can check the results. We will write our reports in a way that no one can work out that your child took part in the study.

What are my choices about how my child's information is used?

- You can stop your child being part of the study at any time, without giving a reason, but we will keep information about your child that we already have.
- If you choose to stop taking part in the study, we would like to continue collecting information about your child's health from your child's medical records. If you do not want this to happen, tell us and we will stop.
- We need to manage your child's records in specific ways for the research to be reliable. This means that we won't be able to let you see or change the data we hold about your child.
- If you agree to take part in this study, you will have the option to take part in future research using your child's data saved from this study.

Where can I find out more about how my child's information is used?

You can find out more about how we use your information:

- at www.hra.nhs.uk/information-about-patients
- our leaflet available from www.hra.nhs.uk/patientdataandresearch
- by asking one of the research team
- by sending an email to the Data Protection Officer at the Intensive Care National Audit and Research Centre: dpo@icnarc.org

What if there is a problem?

If you have a concern about any aspect of the PICU Platform Trial, you should ask to speak with the Principal Investigator or Research Team at the hospital where your child is being cared for who will do their best to answer your questions. If you remain unhappy and wish to complain formally, you can do this through the NHS Complaints Procedure. Details can be obtained from the hospital or the Patient Advisory Liaison Service (PALS) – please go to www.nhs.uk to find your local PALS contact details. You may also have the right to lodge a complaint with the Information Commissioner's Office (ICO) (www.ico.org.uk).

It is very unlikely that any participants in this research will come to any harm as a result of the study, but we are obliged to mention this possibility.

If you wish to complain about any aspect of the way you have been approached or the way your child has been treated during this study, contact the hospital's PALS for further information. The NHS Clinical Negligence Scheme for Trusts provides full financial liability for harm caused to participants in the study caused through negligence. The Intensive Care National Audit and Research Centre (ICNARC) provides insurance cover for negligent harm caused as a result of the design and management of the study. In the unlikely event of a claim for which negligence could not be demonstrated, you may need to take legal action for which you would need to pay.

To leave the study at any point, you can contact the Principal Investigator (the person leading the study at this hospital) using the details at the end of this information sheet, or by contacting ICNARC on [telephone number] or [email address].

Contacts

Thank you for taking the time to read this information sheet. If you have any questions, please contact us:

**Principal Investigator**	**Research Nurse**
[to be localised]	[to be localised]
