# Supplementary material for: Developing an adaptive paediatric intensive care unit platform trial with key stakeholders: a qualitative study
Source: BMJ Open. 2025 Jan 7;15(1):e085142. doi: 10.1136/bmjopen-2024-085142 (PMC11749188; doi:10.1136/bmjopen-2024-085142)
Supplement: online supplemental file 2 [file bmjopen-15-1-s002.pdf]

Study to develop a platform trial for critically ill children

Topic guide for parent/guardian focus groups

Introductions

Opening presentation by facilitator

1. **What are your initial thoughts about a platform trial for PICU?**

Show informed consent and research without prior consent slides and video

1) Consenting process

Explanation about what research without prior consent is. Give examples of how it has been used in multiple studies.

2. **What do you think about doing the Platform Trial without asking parents/guardians or children for their consent first when children are very sick?**
3. **The doctor will explain the research to parents/guardians when their child is a little bit better, but children may still be too poorly to be involved in discussions about research and make a decision about whether to be part of the study. Do you think it is OK for parents/guardians to make decisions for children about their involvement in the Platform Trial if they are still poorly?**
4. **Do you think that children and young people should be given information about the Platform Trial when they are feeling better? Do you think they would want to know? How do you think your child would feel if you told them that they had been part of a study when they were last in hospital?**
5. **When do you think is a good time to approach parents and children and give them the information about the Platform trial?**
6. **Can you think of a different way for children to be given information about the study other than giving an information sheet to parents/guardians?**
7. **Could you describe the possible benefits you would expect from taking part in the platform trial? Prompt: any concerns?**

2) Participant information

Refer to PIS sent prior to the focus group. Explain this is an example and how the information will change depending on what areas/domains are being investigated at a particular point in time. Point out the sections that will remain similar.

8. **What do you think about the format of the Participant information Sheet?**
9. **Could the information sheet be improved in any way?**
10. **Some trials use videos or animations to help describe the research. Do you think this would be helpful for the platform trial? What information do you think is most important to include?**

3) Outcome measures

Explanation about what outcome measures are. Share slide showing pre-defined list of outcomes.

11. **Are there any outcomes missing from this list that you think we should consider for the platform trial?**
12. **Which outcomes do you think are most important?**
13. **Please can you rank the outcomes in order of most important to least important?**

4) Initial trial domains

Show slide with domains suggested by doctors and identified from other studies for this platform trial

14. **What are your thoughts about these initial domains? … Do you have any questions about these domains?**
15. **Are there any domains that are missing?**
16. **Are there any domains or types of medicine/interventions that you think should not be included in the platform trial?** *(If yes, why?)*
17. **Which domains do you think are important to include in the platform trial?**
18. **How many different domains do you think is acceptable for children to be included in at any one time within this proposed trial?**
19. **Do you think that parents should be given the option to opt their child out of any domains so that they didn't have to be included in all of them? *(If yes, explore why).***
20. **Would you be happy if new treatments that aren't usually used in paediatric intensive care, or new ways of caring for children, are used in the PLATFORM trial? (these would not be brand new, untested treatments and may be used in other settings such as in NICU (tiny babies) or adults (*If yes,* how many 'new' treatments are acceptable in one area?)**
21. **Who do you think should be involved in making the decisions about adding a new domain to the platform trial?**
22. **Is there anything you think the research team should think about, or particular people they should speak to, when adding a new domain or intervention into the platform trial?**

Overall

23. **Thinking about everything we have discussed today; do you think it would be acceptable to conduct the proposed PICU platform trial?**
24. **What advice would you give to the researchers who will be approaching families about the platform trial in paediatric intensive care units?**
