# Supplementary material for: Developing an adaptive paediatric intensive care unit platform trial with key stakeholders: a qualitative study
Source: BMJ Open. 2025 Jan 7;15(1):e085142. doi: 10.1136/bmjopen-2024-085142 (PMC11749188; doi:10.1136/bmjopen-2024-085142)
Supplement: online supplemental file 3 [file bmjopen-15-1-s003.pdf]

P I C U
Platform Trial

OUTCOMES

- An outcome measure refers to **‘what’** should be measured in a research study to find out whether a treatment helps to make children better.

- Studies often have a number of outcome measures to determine whether a treatment works. Some are measured during a child’s stay in hospital, whilst others are measured either at the end of their hospital stay or when they have left hospital.

- Researchers or doctors often suggest outcomes that they think should be measured in a research study. However, they do not always know what it’s like to be a sick child or outcomes that are important to their family. That is why it’s important we ask children and their family what outcomes they think a research study should measure to decide whether a treatment is effective.

- Below is a list of outcomes that might be useful to measure for this proposed trial. We want to know which ones you think are important and if any are missing.

- It’s not a test! We just want to make sure we include outcomes that are important to children and their parents/guardians.

The number of days the child spent in the Paediatric Intensive Care Unit (PICU) within a certain time period (usually period of days/months).

The number of times the child was admitted to PICU during the hospital stay.

Overall length of the child's hospital stay(s).

Readmission to PICU/hospital within a certain time period (usually a period of months/years).

The number and type of child's organs (e.g. lungs, kidneys, etc.) **that required support while in PICU/hospital** (e.g., ventilation, dialysis).

The number of days a child's organs required support within a certain time period (usually a period of days/months).

The reason why a child's organs needed support.

The type of support (e.g., non-invasive ventilation) **or treatment/medication** (e.g., blood transfusion, steroids, antibiotics, sedatives, analgesics) **given, and when.**

The number of times (and when) diagnostic tests were carried out within a certain time period (usually a period of days/months).

Symptoms of medical condition(s), disease(s), or infection(s) (e.g., presence of symptoms such as fever, pain, or altered levels of consciousness).

New medical condition(s), disease(s), or infection(s) (diagnosed or proven).

Adverse events (a general term used to describe things that don't go as planned but aren't included in other outcomes. e.g., the number of health complications that occurred as part of a child's illness (or from a treatment) and the time it took for the child to recover from these).

Survival (whether the child survived to a certain time point (usually time point at months/years) or to a specific event (e.g., hospital discharge)).

Child quality of life (how the child's health may impact upon their health, comfort, happiness and ability to participate in daily life – the impacts could be longer term, e.g., reduced physical (mobility), cognitive (brain) and emotional (feelings) functioning when discharged home).

Family quality of life (how the child's health may impact upon family members' health, comfort, happiness and ability to participate in daily life).
