# Supplementary material for: Developing an adaptive paediatric intensive care unit platform trial with key stakeholders: a qualitative study
Source: BMJ Open. 2025 Jan 7;15(1):e085142. doi: 10.1136/bmjopen-2024-085142 (PMC11749188; doi:10.1136/bmjopen-2024-085142)
Supplement: online supplemental file 4 [file bmjopen-15-1-s004.pdf]

Slide shown and explanation provided to participants about what domains are in a Platform trial

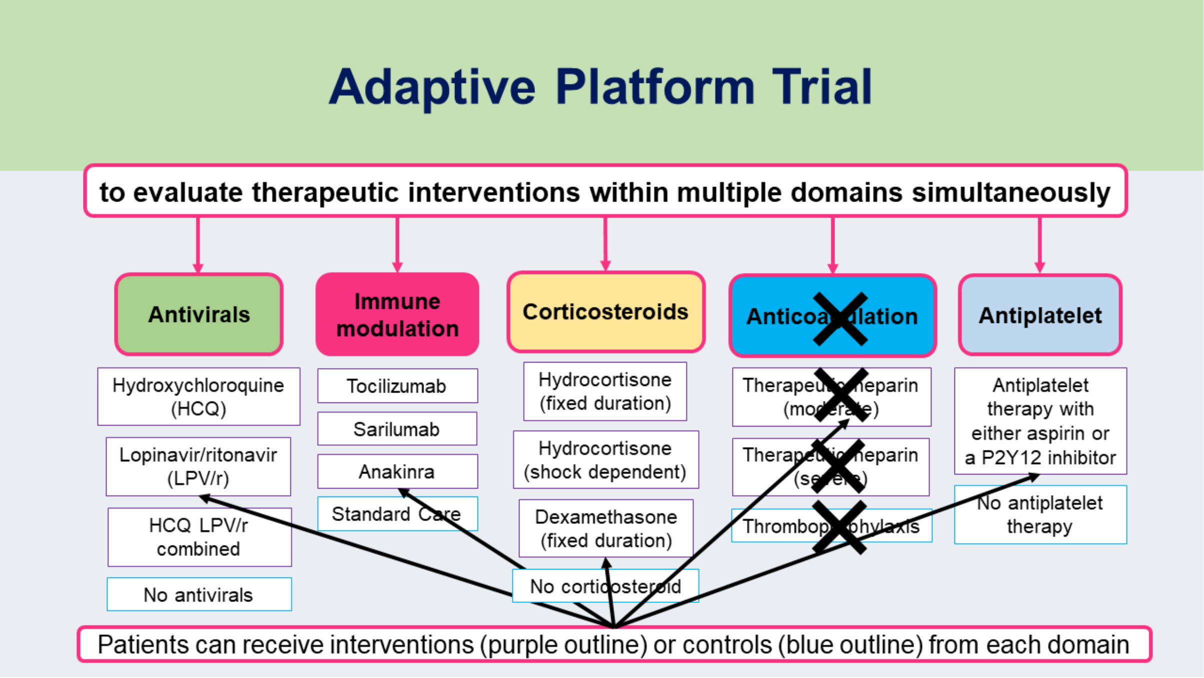

Platform trials are <u>one</u> trial that can have different domains/categories of treatment *(point)* and interventions/control (e.g., medicines, ways of working and/or medical equipment) *(point)* within those domains to be tested at the same time within a single study. So, the domains *(point)* and interventions within them *(point)* run at the same time and new domains and interventions can be added in (or stopped) as the trial goes on.

One international COVID adaptive platform trial (REMAP-CAP – 15 countries) had 5 domain areas - categories of treatment - (*point to domains)* and various interventions *(point)* that might not typically be used within these domain areas *(point to interventions)* that were added or removed over time.

Platform trials are often used in situations where there are multiple potential treatments for a disease and where it is important to find out which treatments are most effective as quickly and efficiently as possible.

Platform trials are adaptive because:

- Multiple questions can be evaluated simultaneously
- New questions/domains *(point)* and interventions can be substituted into the trial or stopped *(point)* like the Therapeutic Anticoagulation Domain in REMAP-CAP*, when we learn what works *(click)*, what doesn't *(click)* and why as the trial progresses *(when sufficient data have been accrued to answer initial questions, rather than when a pre-specified sample size is reached)*
- The trial can be open-ended, meaning that it does not have a fixed end date.
- Patients who are entered into the trial later can be randomised to treatments that are more likely to be beneficial

Traditional trials are not able to do this. By testing multiple treatments within a single trial, platform trials can help paediatric intensive care research because researchers are better able to identify the most effective treatment (and any interactions between treatments) for children and young people more quickly and with fewer patients and there is less burden for children and their families.
