# Supplementary material for: Developing an adaptive paediatric intensive care unit platform trial with key stakeholders: a qualitative study
Source: BMJ Open. 2025 Jan 7;15(1):e085142. doi: 10.1136/bmjopen-2024-085142 (PMC11749188; doi:10.1136/bmjopen-2024-085142)
Supplement: online supplemental file 6 [file bmjopen-15-1-s006.pdf]

Illustrative quotations for the delivery of PICU-Platform trial information in different formats

Format of information	Illustrative quotations
Participant information sheet/leaflet	*'It's [the PIS'] quite a lot to go through. I think, in the situation when you're in a hospital with your child, it is quite a lot to take in. But I think, it is much better for somebody to go through it with you. I don't think you can just be handed the sheet'* (P2, Mother, FG2).<br><br>*'I think digital copies are really good, but I'm quite old school and I would like a proper hard copy to look at and go back to. I would probably be making notes and that kind of thing. So, I definitely would prefer a paper copy'* (P2, Mother, FG2).<br><br>*'Given that parents are very much in a state of shock, I think having the paperwork to go back [to] and mull over is really important. So, I think just ensuring that there is … [someone] around that can sit down and chat with you about it, a week later, after you've consented, to clarify or whatever, is important. Because people might have questions that come up later'* (P6, Mother, FG1).<br><br>*'Send a leaflet [PIS] through the post'* (YP8, Male, FG1).<br><br>Whilst young people recommended giving parents only the information about the domains that pertained to their child, parents recognised that '*logistically [this would be challenging], … there could be hundreds of combinations … somebody could be included in two or three, and which two and which three. You could be making millions of different leaflets'* (P2, Mother, FG2). Parents suggested having a page for each domain in the PIS so that they can skip to the page(s) relevant to them '*because then you don't have to skim through the whole- you know, if you look at the title and you're, like, "Oh, that doesn't apply," you can just skip that whole page. You don't have to then read through that to find the next bit that does apply to your child'* (P2, Mother, FG2).<br><br>Create information sheets for Local Authorities: *'Some of the more vulnerable, disadvantaged children that we look after, so looked-after children are often not included into research. I think we need information sheets for local authorities, which we don't do currently…. They don't receive any teaching about clinical research in hospitals. I think we do need to have information sheets for local authorities because we exclude them all the time'* (PICU staff, FG1).
Advertisements (posters; in the Welcome Pack) on PICU	*'I think you're going to have to have it quite well advertised, if you like, on the unit because the research staff, as much as we love being here, are not here 24 hours a day and the nursing staff on the unit will be responsible for randomising for each of those arms of the study. And I think it's got to be almost something like in the old-fashioned welcome pack thing that when they arrive on the unit, this is going on on the unit, "Your child will receive different care, somebody will come and talk to you at some point." I think there's got to be some sort of pre-emptive thing. We've had interesting comments with the [name] Study that's running at the moment, in that we've got posters everywhere around the unit, and very often we'll go to the families and they know that their kid's on [treatment] and they'll say, "We were expecting somebody to come and talk to us about this"* (PICU staff FG5).
Study website	*'You can have all of this [information] on a website, you can have like a QR code and a simple leaflet, and it can use the QR code to go on to a website where they can find all this information'* (YP7, FG1).
Videos/Videos and PDF files on Social Media	*'I don't know who this would be, but if you have somebody that, kind of, you know like when people do online training but not live, you know, pre-recorded, I wonder if you could have a doctor or a nurse, or somebody involved in the trial, almost saying what they would say to*

	you in person. I think I do find that quite helpful to… hear somebody say it. Yeah, that might be helpful' (P2, Mother, FG2).<br><br>*'Good suggestion from P4, Mother, FG2 about [having a link to] a social media group with videos explaining the information sheet'* (Researcher 2). '*You just open it up and then you've got that video at hand, as well, that would be good'* (P4, Mother, FG2). '*We could put the videos like this onto'* (Researcher 2) *'TikTok, Instagram … Facebook'* (P2, Mother, FG2), *'Snapchat… and YouTube'* (P4, Mother, FG2).<br><br>*'The thing about a video is, you could play it again when you get a bit stressed, or you're thinking about something you're not sure about, and miss the next part. So, you think, "What did he say?" … 'visual information [is] easier to take in and make sense of quickly'* (P7, Mother, FG2).<br><br>*'If you send people a PDF of, this is the overall information, and then just have seven links [to the domains] and tell them which ones are applicable to them, and they can view 30-second video on YouTube that talks them through it, in more detail'* (P6, Father, FG2).<br><br>*'Videos good – good for the visual learners'* (P17, Male, FG2).<br><br>*'Different languages for different nationalities'* (P8, Father, FG2).<br><br>*'That's the good thing about YouTube, you could just click to a different one so it's in your language'* (P5, Mother, FG2).<br><br>Young people from both focus groups also suggested that an *'animation or video… especially for those maybe English is not their first language… might be easier to see it visually than through one of these leaflets [PIS]'* (YP7, Male, FG1).<br><br>*'I think children are more visual learners. I don't think reading will really help them understand about the trial. I think a video's way better'* (YP8, Male, FG2).
Face-to-face by an '*approachable, friendly'* person (P2, Mother, FG2) who has a *'compassionate manner'* (P4, Mother, FG2).	*'It is much better for somebody to go through it with you. I don't think you can just be handed the [PIS] sheet. But I think I would like someone to go through it with me, explain it all. I'd like to probably know about the whole thing, the bigger picture, but then take the information sheet with me to go over it, or to re-read and digest. Then, maybe, ask more questions once I've read it myself. I think, if someone is going through it with you first, then, yeah, I'd be quite keen to know about the whole thing and what parts my child may or may not be included in'* (P2, Mother, FG2).<br><br>*'It's nice to have things in different formats. So, if somebody discusses it, you've got something in your hand, and if you want to go and do your further research you can do that too'* (P5, Mother, FG2).
Having the contact details for research staff	*'If somebody could be accessible to talk about it after. Because immediately when they say it, it's all, "Oh, okay." Then, a few minutes later, or a little while later, how do you get in touch with those people to ask your questions and say, "I wasn't sure what you said, can you please explain?"'* (P5, Mother, FG2).
Reflection time before re-approaching parents	*'I think, just that reflection time, really. I think [Child] went straight into a trial that we didn't know about at the time… They came and talked to us about that, afterwards. I think it was, maybe, the day after we got there. Then, they did that, and they gave us a bit of reflection time and said, "We'll come back to you, again." Because you forget, sometimes, to chase things up. So, that is a good thing, I think'* (P7, Mother, FG2).<br><br>*'Just ensuring that there is the resource around that can sit down and chat with you about it, a week later, after you've consented, to clarify or whatever, is important. Because people might have questions that come up later'* (P6, Mother, FG1).
