# Supplementary material for: Developing an adaptive paediatric intensive care unit platform trial with key stakeholders: a qualitative study
Source: BMJ Open. 2025 Jan 7;15(1):e085142. doi: 10.1136/bmjopen-2024-085142 (PMC11749188; doi:10.1136/bmjopen-2024-085142)
Supplement: online supplemental file 7 [file bmjopen-15-1-s007.pdf]

Suggested edits to outcomes for the PICU-Platform trial

Pre-defined outcome	Edits suggested
**Number of days spent in PICU within a certain time period** (Length of PICU stay)	Change wording to *'The number of days that the child requires level 3 acute critical care'* (measured to the time/day that child is fit for discharge) to account for any non-clinical delays in discharge (PICU staff, FG3).
**Overall length of the child's hospital stay(s)**	Similar to edits suggested for 'Length of PICU stay', change to 'Time fit for hospital discharge' to account for service resource utilisation and children still being *'so weak'* (PICU staff, FG3).<br><br>Linked to 'Child quality of life' - Change to ***'length of time to recovery*** *or discharge, or something like that, after PICU. The only reason I said it was because … our son went from PICU, not home, but back to a ward… He was* ***still really ill when he left PICU, but just not PICU ill. It took a long time for him to get back to normality after that'*** (P1, Father, FG2).
**The number and type of child's organs** (e.g. lungs, kidneys, etc.) **that required support while in PICU/hospital** (e.g., ventilation, dialysis).	*'Maybe length of stay on the ventilator, which isn't specifically there?'* (PICU staff, FG3).<br><br>*Linked to 'Adverse event' - 'Major morbidity in terms of permanent loss or damage of an organ like permanent renal failure or a major disability'* (PICU staff, FG3).
**The number of days a child's organs required support** within a certain time period	Change to 'Length of time on advanced organ support (delivered in PICU/PCC only) e.g., invasive ventilation, inotropes'.<br><br>Make composite with mortality (survival) (PICU staff, FG6).
**Symptoms of medical condition(s), disease(s), or infection(s)**	Include pain scores; sedation scores; delirium/withdrawal.
**New medical condition(s), disease(s), or infection(s)** (diagnosed or proven).	Include 'Major morbidity – permanent organ damage or disability', and overlapping with 'Adverse events', iatrogenic complication.<br><br>*'Antimicrobacterial stewardship… - the incidence of multidrug resistant organisms'* (PICU staff, FG3).
**Adverse events**	Include iatrogenic complication/conditions/injuries (health care acquired conditions/injuries), MCAI's etc. (PICU staff, FG3).
**Survival**	Change to Survival *'to home'*, which is important to families, although *'mortality at 30 days … [differentiates] what is a direct consequence of PICU and what isn't'* (PICU staff, FG5).<br><br>Measure PICU mortality.<br><br>Also measure *'survival after PICU discharge, or maybe survival of one year'* (PICU staff, FG6).
**Child quality of life**	*'It's more than what's going to happen once you leave, like how are you going to live after that? Like, if they say two weeks or four months, it doesn't matter as long as your outcome is still the same, you're still going to be able to do what you want to do when you leave hospital'* (YP5, Female, FG1).

	'If there's a better chance of a better quality of life and survival or less side effects, I think that's something you should consider because this child is only a child and it could affect them forever, so I think that's very important to think about' (YP6, Female, FG1).<br><br>Should *'include child **change in function** (loss of function, including cognitive function) and long-term **educational attainment** outcomes via, for example, school data linkage, BUT DIFFERENCES NEED TO BE COMPARED FROM PRE-HOSPITAL ADMISSION TO HOSPITAL DISCHARGE'* (PICU Staff, FG3), so cognitive function AND educational attainment.<br><br>Previously healthy children (PICU staff, FG1).<br><br>*'Ongoing medical burden of what they're left with when they're discharged from hospital, like how many therapies they're on, any medications they're taking and whether they've got any impact of things like post-PICU syndrome, because I know that's something that's coming more to the fore of the long-term, particularly neurodevelopmental side of what we're doing, outcomes for that, as well as quality of life, the actual measurable side effects of being in PICU as well'* (PICU staff, FG6).<br><br>*'Education and awareness of condition to others (church / school / peers / healthcare workers)'* - *'In child quality of life, is it included the child's condition to the school and how the school staff are informed about it? … Because the attention they get is … completely different when they go to primary school'* (P2, Mother, FG1).<br><br>Include *'mental health … [and] neurodiversity issues… [that] develop as they get older'* (P7, Mother, FG2).<br><br>Parents *'actually want long term'* outcomes (PICU staff, FG3).
**Family quality of life**	Overlapping with child quality of life (YP9, Female, FG2).<br><br>Include *'Family experience of care'* (PICU staff, FG3).<br><br>*'If it causes particularly harmful side effects … that could affect the family as well. It all connects'* (YP5, Female, FG1).<br><br>Include siblings - *'We've got another child. When you say 'child' quality of life, it's like sibling quality of life. I really feel that my older child is missing out on a lot because we don't spend as much time with her. We dump her at godparents, to take our youngest to hospital. I feel that sibling quality of life… For me personally, I feel almost like I don't … give enough to my oldest child. I do try, but unfortunately that is another measure, that I feel she doesn't get enough'* (P4, Father, FG1).<br><br>To include mental health impact (PTSD) if you were looking at a coping intervention for parents or something (PICU staff, FG3).<br><br>Include economic cost to the family; relationships between staff and family; patient and family experience; and previous parent satisfaction.<br><br>Parent mental health (long term regret about whether they made best decision to participate in the trial *'if it was the worst outcome'* (P4, Mother, FG2) and their child dies) (P2, Mother, FG2).
