# Supplementary material for: Developing an adaptive paediatric intensive care unit platform trial with key stakeholders: a qualitative study
Source: BMJ Open. 2025 Jan 7;15(1):e085142. doi: 10.1136/bmjopen-2024-085142 (PMC11749188; doi:10.1136/bmjopen-2024-085142)
Supplement: online supplemental file 8 [file bmjopen-15-1-s008.pdf]

Ranking of outcomes for all participants

**Ranked**	**PICU staff, FG3** (Outcomes FG at PCCS-SG meeting) **(n=9)**	**PICU staff, FG5 (n=9)**	**PICU staff, FG6 (n=4)**	**Young person FG1 (n=9)**	**Young person FG2 (n=9)**	**Parent FG1 (n=7)**	**Parent FG2 (n=8)**
**1st (Most important outcome)** (Score 16)	Length of PICU stay<br>Duration of organ support	Length of PICU stay	Length of PICU stay	Adverse events<br>Survival<br>Child quality of life<br>Family (including sibling(s)) quality of life	Survival	Adverse events	Survival 'to home'
**2nd** (Score 15)	Survival	Duration of organ support<br>Child quality of life	Duration of organ support		Child quality of life	Child quality of life<br>Family quality of life	Adverse events
**3rd** (Score 14)	Child quality of life	Survival	Survival<br>Child quality of life		Family quality of life<br>Cost/Health economic outcomes	Symptoms of medical condition(s), disease(s), or infection(s)	Child quality of life
**4th** (Score 13)		Number of times admitted to PICU during the hospital stay	Re-admission to PICU/hospital within a certain time period			Type of intervention (support / treatment / medication) given, and when	Length of PICU stay
**5th** (Score 12)		Overall length of hospital stay	Overall length of hospital stay			Number and type of child's organs that required support while in PICU / hospital	Overall length of hospital stay

Ranked 6th (Score 11); 7th (Score 10); 8th (Score 9); 9th (Score 8); 10th (Score 7); 11th (Score 6); 12th (Score 5); 13th (Score 4); 14th (Score 3); 15th (Score 2); 16th (Score 1)
