# Supplementary material for: Developing an adaptive paediatric intensive care unit platform trial with key stakeholders: a qualitative study
Source: BMJ Open. 2025 Jan 7;15(1):e085142. doi: 10.1136/bmjopen-2024-085142 (PMC11749188; doi:10.1136/bmjopen-2024-085142)
Supplement: online supplemental file 9 [file bmjopen-15-1-s009.pdf]

Subgroup populations who might influence outcomes

Stratum	Illustrative quotations
**Cardiac/non-cardiac surgical population**	*'So, I know from cardiac, for many years, Midazolam isn't the ideal drug of choice. So, it's not something we would- We do use it occasionally. But obviously, we've gone away from using that, really, because it's not good on the heart'* (PICU staff FG6).<br>*'Blood transfusions, from my perspective, cardiac intensive care, doing ECMO and things, it's very different from deciding whether or not you're just going to top up an anaemia, or at what point do you do that, because it can vary depending on what the child's condition is'* (PICU staff FG6).<br>*'Certainly, things like temperature, you wouldn't get much buy-in, in terms of equipoise, for managing different temperatures in cardiacs. Transfusion, I'd imagine you'll get some input from the PETRA study'* (PICU staff FG6).<br>BUT staff would want cardiac patients included: *'I work in a mixed unit. We're about 50/50 split cardiac, including ECMO, and general. Those populations are managed very differently, even on our unit. So that my key concern would be about making sure that the cardiac population for our unit are not completely excluded from this and are able to join in'* (PICU staff FG6).
**Age**	*'I think intuitively the new- so the below one-month-olds are probably interesting to look at separately to see, especially because a lot also just show a lot of the targets in them. For example, if you think about transfusion or stats, or whatever target you're setting, they might have quite different targets than the rest of the population'* (PICU staff FG5).
**Planned/unplanned admissions**	*'Maybe you could cover two or three of the strata just by … [having] planned and not planned'* (PICU staff, FG1) and *'that will separate cardiac and non-cardiac'*, too (PICU staff, FG1).
**Children with traumatic brain injury**	*'Patients [with] … traumatic brain injury, which seems physiologically, for example, they're more prone to hospital-acquired infection… And also, again, their quality of life and outcomes might be influenced, not by the intervention, but by their disease'* (PICU staff FG5).<br>*'The way that they're managed, so from a temperature management it's completely different… The sedation, we would use different types of sedation with them, perhaps. Fluid management is probably different than it would be for- depending on how bad they are'* (PICU staff FG5).
**Children with cancer**	*'Oncology is a good example, isn't it, for where you might be anxious about treatment for therapy for fever or blood transfusion targets'* (PICU staff FG5).<br>*'Oncology … are higher, obviously, in mortality'* (PICU staff, FG1).
**Children in receipt of palliative care**	*'Most outcome measures are going to include mortality somewhere, so if someone is actually on a palliative care pathway you can probably say they shouldn't go in'* (PICU staff, FG1).<br>*'A big proportion of… patients who are on a palliative care pathway… end up having interventions discontinued or withdrawn on the basis of comorbidities'* (PICU staff, FG1).
**Children with multiple morbidities**	*'Patients … with lots of comorbidities, and I don't know how you're going to define that, … [are] going to have longer dependencies, not because of their illness'* (PICU staff FG5).<br>*'Quite a few [children] … with comorbidities… we may end up not continuing to support'* (PICU staff, FG1).

Note: Staff said that there will be some overlap of patients across domains
