# Supplementary material for: Developing an adaptive paediatric intensive care unit platform trial with key stakeholders: a qualitative study
Source: BMJ Open. 2025 Jan 7;15(1):e085142. doi: 10.1136/bmjopen-2024-085142 (PMC11749188; doi:10.1136/bmjopen-2024-085142)
Supplement: online supplemental file 10 [file bmjopen-15-1-s010.pdf]

PICU Staff recommendations for governance

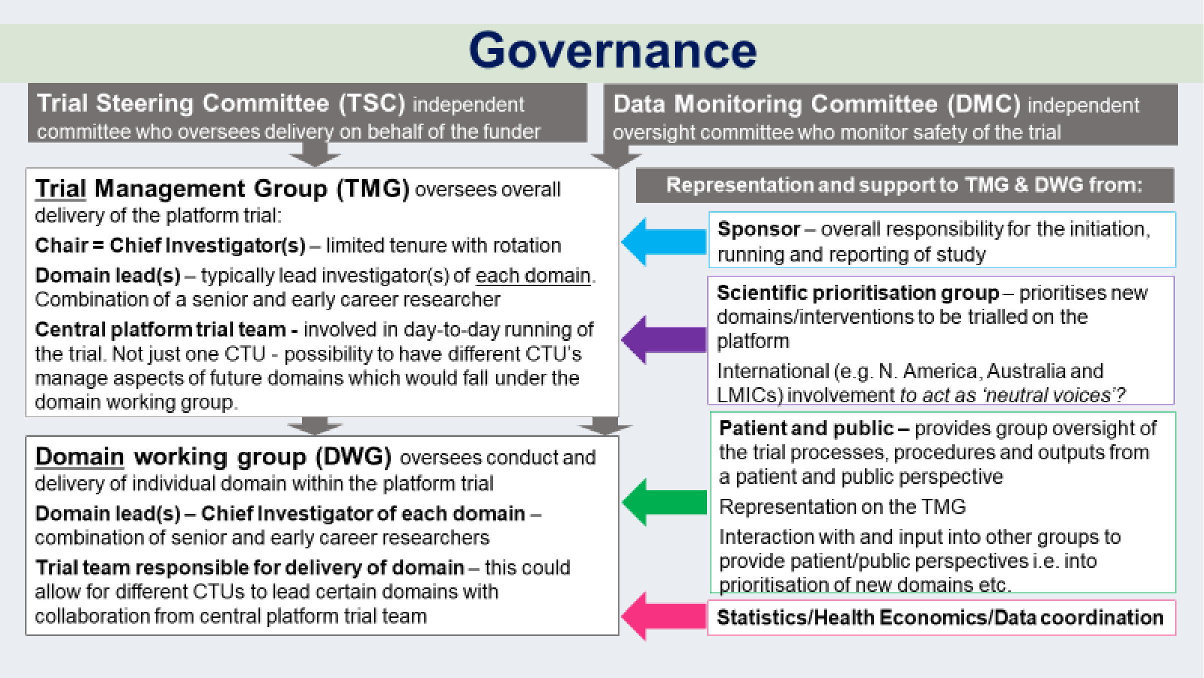

PICU staff suggestions for the structure of the PICU-Platform trial

Illustrative quotations for TMG	A chief investigator (rotating chair with limited tenure – timeframe to be decided) - to *'keep the TMG refreshed… [as each domain might take] four or five years'* (PICU staff, FG4).
	A central platform trial team *'involving different CTUs (clinical trial units), because obviously it will become quite a large trial'* (PICU staff, FG4).
	There *'absolutely needs to be somebody from stats [statistics] … and also, patients and public … representation [… young persons and parents…] sitting on'* the TMG, DWGs and Scientific Prioritisation Group (SPG) (PICU staff, FG4) *'for neutrality and focus on patient outcomes'*, to *'prioritise how… [to] add domains so that it's fair for people, so that it's not a bit of a club?'* (PICU staff, FG4) and ascertain, *'Is it an important question? Do patients think it is? What does this community think?'* (PICU staff, FG4).
	There should be a scientific prioritisation process which involves international research active PICU staff from, for example, *'Australia, New Zealand, North America and potentially also lower- and middle-income countries… who can't be funded by this, so therefore have less bias perhaps'* (PICU staff, FG4) to act as *'neutral voices for non-biased contributors'* (PICU staff, FG4).
	PICU staff also mentioned the importance of keeping *'those of us who are out in the units… [who] are actually going to be doing some of this research on the ground, or they are going to be looking after the patients, filling in the forms… engaged as well'*.
	One staff member suggested ensuring that all paediatric critical care staff (PCCS) are involved in decision making *'because PCCS-SG is a self-selecting group, there are a lot of members of PCCS who may not actively be involved in PCCS-SG who might have quite strong opinions'* (PICU staff, FG5).
	PICU staff said that the process of set up and governance should be inclusive of all clinical trial units. Regularly assessing research within the platform trial will help to ensure that processes and structures are correct:

	'Thinking about assessing it on a regular basis, as well, to see if this is going the way we want it to go. Something around that because this is all going to be new for all of us' (PICU staff, FG4).
Illustrative quotations for DWGs	Domain lead(s) (split between an early career and senior role researcher - *'then potentially only one of them would need to attend the meetings if people were stretched, because often people are stretched'* (PICU staff, FG4).
	Staff suggested that Domain Working Group (DWG) leads would have representation on the TMG and would be responsible for securing funding. DWG members would be responsible for the conduct and delivery of individual domains within the platform trial, including *'getting sites interested'* (PICU staff, FG4).
	Having *'a staff nurse representative …the person at the bedside… [on the TMG, DWGs and Scientific Prioritisation Group (SPG)] to advocate for … [patient] needs and get a better'* buy-in to the trial and embedding findings in practise.
	To have a qualitative evaluation or study within a trial (SWAT) within DWGs (PICU staff, FG4) because '*it does feel like we are weighting everything towards trials when actually we are recognising the importance of much more mixed methods stuff… It is that feeling of, are we going to create a two-tier only trials count? I thought we were moving away from that bit?'* (PICU staff, FG4).
	Staff said that *'people [will] come in with ideas for domains as time goes by'* and emphasised the importance of having *'arrangements in place for people to be credited for their intellectual input into the work'* (PICU Staff FG6):<br><br>*'How to ensure the recognition of the Domain Working Group… making sure it's not just the Chief Investigator first author on every publication. I guess we probably see it as the domain leads take the lead on those publications and it's the Domain Working Group who writes it on behalf of, but that would need agreement'* (PICU Staff, FG4).
