# Supplementary material for: Developing an adaptive paediatric intensive care unit platform trial with key stakeholders: a qualitative study
Source: BMJ Open. 2025 Jan 7;15(1):e085142. doi: 10.1136/bmjopen-2024-085142 (PMC11749188; doi:10.1136/bmjopen-2024-085142)
Supplement: Supplementary file 11 [file bmjopen-2024-085142.reviewer_comments.pdf]

PEER REVIEW HISTORY

BMJ Open publishes all reviews undertaken for accepted manuscripts. Reviewers are asked to complete a checklist review form and are provided with free text boxes to elaborate on their assessment. These free text comments are reproduced below.

ARTICLE DETAILS

Title (Provisional)

Developing an adaptive paediatric intensive care unit platform trial with key stakeholders: a qualitative study

Authors

Mitchell, Tracy Karen; Menzies, Julie C.; Ramnarayan, Padmanabhan; Gould, Doug William; Deja, Elizabeth; Marsh, Shelley; Ainsworth, Jennifer; Preston, Jennifer; Sedgwick, Hannah; Tibbins, Carly; Mouncey, Paul R.; Peters, Mark J.; Woolfall, Kerry

---

VERSION 1 - REVIEW

Reviewer 1

Name Lin, Ying Ling

Affiliation University of Toronto, Institute of Biomaterials and Biomedical Engineering

Date 07-Mar-2024

COI None.

Dear authors,

Many thanks for the opportunity to review this manuscript. The PICU in question highlights small differences across ICUs. For instance, the ICU where I conducted my research was already divided into PICU and Cardiac ICU, so this separation was already in place and could be a non-issue at some hospitals. In general, I would also like to see if there is a chance to incorporate physiological data together with time of intervention into the collected data sets. This could help trace back any outcomes down to the way the interventions are administered.

This study was very interesting to read. I have only minor comments which I list below:

Page 3, line 13: The acronym PPIE did not seem to be defined earlier and seems to be the first instance in the manuscript. Please define at this point.

Page 3, line 16: Regarding a higher number of participants (86) took part in this study, to what other study is this compared to? Perhaps a reference study with the lower number or restricted types of participants should be added.

Page 3, line 24: Was language the only barrier to child/parent participation? Could there have been cultural barriers to parent participation?

Page 3, line 46: Please elaborate on type of patient data. Are these continuously monitored data, including from the physiological monitor, the ventilator, infusion pumps medication and rate, medical record notes? If O2 purity is one of the research questions, can other dynamic interventions be recorded to ensure those are not confounding with the main intervention.

Page 5, line 52: Since coding was done by one person with check from another author how can coding consistency be ensured independently? Was there an exercise of independent coding before all transcripts were coded by a single person?

Page 9, line 20: I thought these results were interesting across participant types. Could this paragraph be interpreted in the discussion? Why do staff prioritize fluids and sedation, while parents prioritize diagnostics and NIV. And why do young people prefer to know about interlinked goals like fluid and temperature management?

Page 12, line 60: Perhaps replace “participants” with “participation.”

Best regards,

Ying Ling Lin, PhD

**Reviewer**	**2**
**Name**	**Donnelly, Peter**
**Affiliation**	**The Royal Hospital for Children, Paediatric Intensive Care Unit**
**Date**	**10-Mar-2024**
**COI**	**I have no competing interests.**

The authors should be commended on this important piece of work. The submitted manuscript is robust and thorough. The prose is well-written and easy to follow. Platform trials are an important consideration for future research within PCC and this study is a fantastic start. The inclusion of young people and families to inform study protocol is fundamental. Well done. I have no suggested changes to this version.

**Reviewer**	**3**

**Name**	**Shaw, Rachel**
**Affiliation**	**Aston University, Institute of Health & Neurodevelopment**
**Date**	**27-Mar-2024**
**COI**	**I have no competing interests**

The manuscript describes an exploratory study with key stakeholders of PICU about setting up a platform trial. It is a little unclear what the function of a platform trial is and so I think the authors need to make that much clearer from the outset – both in the abstract and the start of the introduction.

The work is of a high quality, using appropriate methods, and is very transparently reported. The goal is laudable and will certainly benefit future research.

I don't think the authors need to do anything much for this to meet publication standards. Below are some comments for consideration which may require minor changes. I really enjoyed reading the paper and look forward to seeing it in published form.

Abstract

It's not that clear from the abstract what the purpose of the platform trial is. A brief sentence in the background could simply describe that before going on to describing how it was received. It feels like the paper is about working together with children, parents/carers & staff in the development of a trial, but that isn't too clear at this point. It needs to be clearer so that readers know from the outset what the paper – and indeed platform trial – is aiming to achieve.

This becomes clear in the "what is already known" section but I think it needs to be clearly stated in the abstract.

The strengths & limitations section is great – honest & clearly identifies the challenges with this kind of inclusive, collaborative research.

Main body

It's refreshing to read such a transparent description of how research is set up and how researchers set up their stakeholder groups.

I like the reference to information power rather than data saturation, which is only applicable to grounded theory studies. I'm pleased to see the concept of information power being used as a more flexible concept.

I am not surprised to read that the timing of the approach to take part in research is central to parents. It's critical for the research obviously, but parents need to be able to process material to make an informed decision. I'd think that consent taking training and the notion of continual assent taking is required during these meetings.

Generating different formats for PISs is long overdue in my opinion. I'd welcome further work on testing out different formats and whether any aspects of the information on our HRA consent templates can be excluded from children's consent forms or consent forms more generally while still maintaining an appropriate level of informed consent.

Most readers are likely to understand this comment, but a footnote about the use of these drugs might be required for a less medically knowledgeable reader: Dexmedetomidine and Midazolam.

Discussion

The issue relating to whether parents should be consulted before children are asked is a tricky one. Perhaps there's room to consider age-related decisions, although of course legally, parents are required to give consent for children aged under 16. Perhaps it's worth covering some of these issues a little or at least referring to the HRA's legal position on research involving children versus what might feel like what is morally good/right for the child from a clinical expertise perspective.

https://www.hra.nhs.uk/planning-and-improving-research/policies-standards-legislation/research-involving-children/

You're likely aware of this, but the concepts discussed here in relation to establishing a practitioner-patient relationship could be relevant to the issues raised here around negotiating research participation and informed consent.

https://pubmed.ncbi.nlm.nih.gov/20552281/

it's encouraging as a qualitative researcher myself to see that data are not shareable because they come from a small, potentially identifiable, pool, which makes it impossible to fully protect participants' anonymity. This is an issue we often grapple with so I think seeing it in a reputable journal like this will be helpful to others working with similar groups.

VERSION 1 - AUTHOR RESPONSE

Please see our point-by-point responses below which include explanations of associated changes to the original article. These are addressed in the order of the paper structure.

**Abstract**	
**Reviewer 3**<br>It is a little unclear what the function of a platform trial is and so I think the authors need to make that much clearer from the outset – both in the abstract and the start of the introduction.<br><br>It's not that clear from the abstract what the purpose of the platform trial is. A brief sentence in the background could simply	A sentence has now been added to the beginning of the 'Objectives' section of the Abstract which clarifies what the function of a platform trial is:<br><br>**Objectives: Platform trials were used successfully in adult populations during the COVID-19 pandemic. By testing multiple treatments within a single trial,**

describe that before going on to describing how it was received. It feels like the paper is about working together with children, parents/carers & staff in the development of a trial, but that isn't too clear at this point. It needs to be clearer so that readers know from the outset what the paper – and indeed platform trial – is aiming to achieve. This becomes clear in the "what is already known" section but I think it needs to be clearly stated in the abstract.	**platform trials can help identify the most effective treatments (and any interactions between treatments) for patients more quickly, and with less burden for patients and their families.** The aim of this qualitative research with young people, parents/carers and paediatric intensive care unit (PICU) staff in the United Kingdom was to inform the design of the first adaptive platform trial for paediatric intensive care.
**Strengths and Limitations**	
**Reviewer 1**<br>Page 3, line 13: The acronym PPIE did not seem to be defined earlier and seems to be the first instance in the manuscript. Please define at this point.	The PPIE acronym has now been defined. Thank you for noticing this.
Page 3, line 16: Regarding a higher number of participants (86) took part in this study, to what other study is this compared to? Perhaps a reference study with the lower number or restricted types of participants should be added.	We have amended the 'Strengths and limitations of this study' section of the manuscript to relate specifically to the methods (please see Editors request/comment above), so this line has been removed.
Page 3, line 24: Was language the only barrier to child/parent participation? Could there have been cultural barriers to parent participation?	There could have potentially been cultural barriers to parent participation, but we do not have data to support this claim so have not been able to include this in this section.
Page 3, line 46: Please elaborate on type of patient data. Are these continuously monitored data, including from the physiological monitor, the ventilator, infusion pumps medication and rate, medical record notes? If O2 purity is one of the research questions, can other dynamic interventions be recorded to ensure those are not confounding with the main intervention?	The answer to this was not known at this early stage of the trial design and was not something we explored with participants. Thank you for raising these questions, which will be addressed within the PICU Platform Trial protocol.
**Introduction**	
**Reviewer 3**<br>It is a little unclear what the function of a platform trial is and so I think the authors need to make that much clearer from the outset – both in the abstract and the start of the introduction.	The following sentences have been added after the sentence, *'An adaptive platform trial is an alternative way of conducting RCTs and is designed to allow multiple research questions and interventions to be tested simultaneously within 'domains'/areas in a single study'* to help clarify what the function of a platform trial is:

	**'An adaptive platform trial allows for pre-specified changes to the trial as it is being conducted, for example, new research questions or interventions can be added in or stopped as the trial progresses. Patient data are examined at multiple time points, which informs these adaptations. Consequently, important questions can be addressed simultaneously'.**
**Methods**	
**Reviewer 1**<br>Page 5, line 52: Since coding was done by one person with check from another author how can coding consistency be ensured independently? Was there an exercise of independent coding before all transcripts were coded by a single person?	The following sentence has edited (bold text indicates text that has been added):<br><br>TKM led the analysis with oversight from KW. **The transcripts were read and reread to note down initial ideas before being imported into NVivo V14 [28], which is the software that was used to** assist the organisation and coding of data, including the domains and outcomes data collected in Poll Everywhere and exported into Microsoft Excel [29]**. Deductive codes were initially identified from the study protocol and interview/focus group topic guides.** Analysis was then broadly inductive, thematic and iterative [30-32]. **KW second coded 40% of transcripts and then checked these against TKM's codes. Transcripts coded before new codes or sub-codes were identified were revisited to ensure that the new codes were representative of the data coded under them. All codes and sub-codes were interrogated by TKM and KW to search for and name themes that would provide a clear and trustworthy account of the data. TKM and KW met regularly to discuss the names and definitions of codes, sub-codes, themes and sub-themes, so that they remained nuanced. The names of themes and sub-themes were agreed by all authors.** A weighted point-based system was used to determine the top prioritised outcomes…
**Findings**	
**Reviewer 3**<br>Most readers are likely to understand this comment, but a footnote about the use of these drugs might be required for a less medically knowledgeable reader: Dexmedetomidine and Midazolam.	The following text in bold has been added:<br>Some PICU staff had concerns about the use of Dexmedetomidine **(an 'α2 adrenergic receptor agonist… used for procedural sedation or anaesthetic premedication in children' [37 (pp. 12)])**, and Midazolam **(a benzodiazepine which is a**

	**common sedative used in children [37])** in the sedation domain as they did not use this drug at their PICU or would not want to use it for children with cardiac conditions.
**Discussion**	
**Reviewer 1**<br>Page 9, line 20: I thought these results were interesting across participant types. Could this paragraph be interpreted in the discussion? Why do staff prioritize fluids and sedation, while parents prioritize diagnostics and NIV. And why do young people prefer to know about interlinked goals like fluid and temperature management?	These data were collected via a voting system so the reasons why staff prioritised the domains of Fluid management and Sedation management and parents prioritised Rapid diagnostic tests and Non-invasive ventilation as being the most important to include in the PICU platform trial were not explicit. Young people, however, implied that initially prioritising interlinked domains such as Sedation management and Non-invasive ventilation would be a pragmatic approach to take.
**Reviewer 1**<br>Page 12, line 60: Perhaps replace "participants" with "participation."	Thank you for noticing this. The word 'participants' has now been replaced with the word, 'participation'.
**Reviewer 3**<br>The issue relating to whether parents should be consulted before children are asked is a tricky one. Perhaps there's room to consider age-related decisions, although of course legally, parents are required to give consent for children aged under 16. Perhaps it's worth covering some of these issues a little or at least referring to the HRA's legal position on research involving children versus what might feel like what is morally good/right for the child from a clinical expertise perspective.<br>https://www.hra.nhs.uk/planning-and-improving-research/policies-standards-legislation/research-involving-children/<br>You're likely aware of this, but the concepts discussed here in relation to establishing a practitioner-patient relationship could be relevant to the issues raised here around negotiating research participation and informed consent.<br>https://pubmed.ncbi.nlm.nih.gov/20552281/	The Gillick competency sentence has been removed from the paragraph given the context is RWPC. The second and third paragraphs of the discussion section have been edited and now read as:<br><br>A RWPC [3, 20] approach was viewed as being acceptable for **time-critical interventions within** the PICU platform trial, as found in other studies [16, 23]. **There may be a need to seek informed consent or assent (e.g., for follow-up data collection), depending on the selection of interventions and outcomes. In line with the Health Research Authority guidance on consent by and on behalf of children and young people [38], practitioners should assess a child's capacity and provide information that is understandable to them even when the child or young person is not deemed competent to make decisions for themselves or when they are not legally required to do so, for example, in a clinical trial of an Investigational Medicinal Product (CTIMP).**<br><br>**Danaher et al (2023) [39] state that effective communication is critical in healthcare settings for building trust,**

	**sharing information and decision-making.** Parents and young people highlighted the importance of providing parents (and where appropriate, children) with age-appropriate trial information materials at an appropriate time and in different formats (such as a face-to-face discussion, pre-recorded video, animation, participant information leaflet, posters, and study website), as recognised in various fields for accessibility, comprehension, engagement and inclusivity reasons [40]. Consideration should also be given to the environment where this information is given. **Our findings suggest that parents should be informed about research participation first and their views sought on how best to tailor and deliver research information for their child once they are stabilised** to prevent unnecessary burden or distress (non-maleficence [41, 42]). **As highlighted by Habermas' (1985) theory of communicative action [43], practitioners should seek to understand parents' and children's preferences, emotions and experiences by using individualised communication.**

We attach a clean and a marked copy (using track change function) of our revised article.

Thank you, once again, for all your work in considering and reviewing our article.
